# A dose-response curve for biodosimetry from a 6 MV electron linear accelerator

**DOI:** 10.1590/1414-431X20154470

**Published:** 2015-05-26

**Authors:** M.M.P. Lemos-Pinto, M. Cadena, N. Santos, T.S. Fernandes, E. Borges, A. Amaral

**Affiliations:** 1 Departamento de Energia Nuclear, Universidade Federal de Pernambuco, Recife, PE, Brasil; 2 Departamento de Biofísica e Radiobiologia, Universidade Federal de Pernambuco, Recife, PE, Brasil; 3 Centro Acadêmico de Vitória, Universidade Federal de Pernambuco, Vitória de Santo Antão, PE, Brasil

**Keywords:** Dicentrics, Calibration curves, Linear accelerator, CABAS, Dose Estimate

## Abstract

Biological dosimetry (biodosimetry) is based on the investigation of radiation-induced biological effects (biomarkers), mainly dicentric chromosomes, in order to correlate them with radiation dose. To interpret the dicentric score in terms of absorbed dose, a calibration curve is needed. Each curve should be constructed with respect to basic physical parameters, such as the type of ionizing radiation characterized by low or high linear energy transfer (LET) and dose rate. This study was designed to obtain dose calibration curves by scoring of dicentric chromosomes in peripheral blood lymphocytes irradiated *in vitro* with a 6 MV electron linear accelerator (Mevatron M, Siemens, USA). Two software programs, CABAS (Chromosomal Aberration Calculation Software) and Dose Estimate, were used to generate the curve. The two software programs are discussed; the results obtained were compared with each other and with other published low LET radiation curves. Both software programs resulted in identical linear and quadratic terms for the curve presented here, which was in good agreement with published curves for similar radiation quality and dose rates.

## Introduction

Biological dosimetry (biodosimetry) is an important method for estimating the dose of ionizing radiation absorbed by a person, and is based on biological endpoints modified after chronic or acute exposure to this physical agent (1). Among several proposed biological endpoints and cellular biomarkers, the assay of dicentric chromosomes in peripheral blood lymphocytes is the most frequently used and is considered as the gold standard (1).

A dicentric chromosome is a radiation-induced aberrant chromosome formed as a result of misrepair by nonhomologous end joining whereby two damaged chromosomes undergo an exchange of material. A number of studies have demonstrated a close correspondence between the yield of radiation-induced dicentrics and the absorbed radiation dose for either *in vivo* or *in vitro* exposures (1,2).

The main advantages of scoring dicentrics for biodosimetric evaluations are their high radiation specificity, low background in nonexposed individuals (0-1 dicentric per 1000 cells), low intervariability, and low detection limits of 0.1 Gy for low linear energy transfer (LET) radiation (e.g., γ- and X-rays) and 0.01 Gy for high LET radiations such as neutrons (3).

Scoring of dicentrics in peripheral blood lymphocytes cultured *in vitro* is interpreted in terms of the absorbed radiation dose with reference to a dose-response calibration curve generated by irradiating blood samples from healthy, unexposed donors with different absorbed doses of a defined quality of radiation. The yield of radiation-induced dicentrics is dose dependent, increasing linearly for small absorbed doses, and quadratically for high absorbed doses of low LET radiation. This is known as the linear-quadratic model. For high LET radiations, the shape of the dose response is linear, with no quadratic term (4,5).

The correct curve-fitting procedure is not trivial because it requires an appropriate weighting of data points through implementation of algorithms and the calculation of dose uncertainty. The latter is usually reported as the 95% confidence interval (CI) and is associated with two components: the distribution of unstable aberrations in the irradiated sample (corresponding to the Poisson distribution or the overdispersion) and uncertainties associated with the calibration curve (6).

To overcome difficulties in implementing algorithms for the data points fitting and for calculation of confidence intervals on the dose-response curves, computer programs have been constructed. For exposure to low LET radiation, the most widely used software packages to fit dose calibration curves are CABAS (Chromosomal Aberration Calculation Software <http://www.ujk.edu.pl/ibiol/cabas/>>) and Dose Estimate (7). CABAS employs the maximum likelihood (ML) statistical method, whereas Dose Estimate is based on the iteratively reweighted least squares (IRLS) method (8,9).

According to the IAEA (International Atomic Energy Agency) (1), each cytogenetic biodosimetry laboratory should generate its own dose-response curves in order to avoid interlaboratory variations, which have been documented in collaborative exercises (10). Such differences arise from multiple reasons such as intrinsic environmental conditions in each laboratory, choice of reagents, handling procedures and equipment, and the level of training for the subjective nature of microscopic identification of unstable chromosome aberrations. Although a laboratory dose estimation based on a calibration curve obtained by another laboratory may be used as a reference, this practice will introduce additional uncertainties (11).

In order to approximate *in vitro*-generated calibration curves as closely as possible to *in vivo* responses, it is important to generate the curves using a wide range of possible absorbed doses involved in the majority of accidental human exposures to ionizing radiation. Since most radiological incidents involve overexposure to gamma-radiation or X-rays, curves for those two low LET radiations should be the first ones established in biodosimetry laboratories (12).

On the other hand, electron linear accelerators (LINACs) are increasingly becoming the most frequently used device in modern radiotherapy departments. LINACs produce a reliable, flexible and accurate radiation beam that can simply be powered off when not in use (13,14). As a result, in developed and developing countries, ^60^cobalt (1.25 MV) sources have been replaced by electron LINACs over the years, increasing the need for a biodosimetry calibration curve more suitable for energy levels higher than 4 MV in order to be prepared for accidents and incidents with LINACs (13,15).

The purpose of this paper was to construct an *in vitro* dose calibration curve for a 6 MV electron linear accelerator using the dicentric assay and to fit the data to both the CABAS and the Dose Estimate programs in order to compare the output of each method. The curve was compared to other curves reported in published studies and obtained with other types of radiation to investigate differences in the linear and quadratic terms for different kinds of radiation energies. Extending the range of radiation qualities for which the dicentric assay has been calibrated is important because of advances in medical treatment with linear accelerators and for increasing the quality of radiological emergency programs.

## Material and Methods

### Ethics

This work was approved by the Ethics Committee on Research Involving Humans of the Health Science Center of the Universidade Federal de Pernambuco, under registration No. 031/09. Blood samples were obtained with written informed consent, and the donor's privacy rights were observed.

### Irradiation conditions

Peripheral blood samples were obtained by venipuncture from nonsmoking healthy male donors 29 years of age and collected in heparinized tubes. Samples were aliquoted into 3 mL syringes and separately irradiated *in vitro*, at room temperature.

The irradiation consisted of X-rays from a 6 MV linear accelerator (Mevatron M; Siemens, USA) at a dose rate of 0.54 Gy/min. Syringes (3 mL) were positioned in a solid water-equivalent phantom (ρ=0.99 g/cm^3^), which simulated soft tissues of the human body. The blood samples were placed in the center of a 15×15 cm radiation field at a source-sample distance of 0.80 m from the radiation source at the phantom. Each blood aliquot was exposed at room temperature to six different radiation doses: 0.25; 0.5; 1.0; 1.5; 2.0; and 3.0 Gy. The low doses (0.25 and 0.5 Gy) were needed in order to determine the linear alpha term, and the higher doses (≥1 Gy) were needed to determine the beta quadratic term. One nonirradiated (0 Gy) aliquot served as a control sample.

### Lymphocyte cultures

After irradiation, the blood was kept at 37°C in a water bath for 2 hours, before setting up lymphocyte cultures. For each culture, 0.4 mL of whole blood was added to 4 mL RPMI 1640 medium supplemented with 0.5 mL fetal calf serum (Cultilab, Brazil) and 0.1 mL phytohemagglutinin (Gibco, Brazil). The cultures were incubated at 37°C in humidified air with 5% CO_2_ for 48 h. These procedures were consistent with guidelines of the International Atomic Energy Agency (IAEA) manual (1).

Colcemid (Sigma, Brazil) was added at the beginning of cell culture at a very low concentration (0.05 µg/mL) in order to arrest cells at first metaphase. Early addition of this mitotic spindle inhibitor prevented excessive chromosome condensation and allowed for metaphase spreads adequate for scoring aberrations. This was the method of choice because it avoided the possibility of cells escaping from the first division, thus eliminating the need for monitoring the cell cycle with bromodeoxyuridine and fluorescence microscopy plus Giemsa staining.

Cell harvesting was carried out by standard procedures. In brief, after hypotonic treatment with 0.075 M KCl, lymphocytes were fixed in a mixture of methanol and glacial acetic acid (3:1). These harvesting and processing methods have been previously established and tested in our laboratory.

### Chromosomal preparations and slide scoring

Fifty microliters of cell suspension was dropped onto a slide humidified in a water bath at 70°C. The slides were then dried by placing them on a metal hot plate, as described by Henegariu et al. (16) with some modifications. Metaphase spreads were stained with 5% Giemsa solution and air-dried.

For scoring, at least 500 complete metaphase cells with 46 centromeres were counted per sample. In addition to dicentrics, the numbers of centric rings, excess acentric fragments and chromosome breaks were recorded. Slides from each culture were scored by three independent investigators using conventional light microscopes (Leica DME 13595, Germany).

### Statistical analyses

Dose-response calibration curves were constructed with the CABAS Software version 2.0 and Dose Estimate software version 4.1.

To determine whether dicentric frequency followed a Poisson distribution as expected for acute X-ray irradiation, the dispersion index (σ^2^/y) and the normalized unit of this index (u) were obtained for each dose using an equation described in the IAEA manual where *N* indicates the number of cells analyzed and *X* is the number of dicentrics detected.

$$u=\left(\frac{\sigma^{2}}{y}-1\right)\sqrt{N-\frac{1}{2(1-\frac{1}{X})}}$$

Dispersion index values close to 1 and *u* values between ±1.96 indicate conformity with the Poisson distribution. Values of *u*higher than 1.96 indicate an overdispersion of data, whereas *u*values lower than –1.96 indicate an underdispersion (1,11).

The goodness-of-fit and the chi-squared tests for homogeneity were performed with CABAS and Dose Estimate software. In order to correlate the dose delivered and dicentric frequency, Pearson's correlation was determined at the 5% or P≤0.05 level of significance.

## Results

After *in vitro* irradiation with X-rays produced by the linear accelerator, a total of 7,871 metaphase spreads were counted, and all unstable chromosomal aberrations found were recorded. Data obtained following exposure to seven different radiation doses are shown in Table 1.

**Table:**
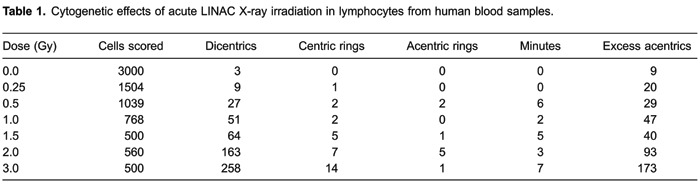

For each type of chromosome aberration, Pearson's correlation coefficients were calculated. Table 2 shows the number of cells scored, the frequency of dicentrics, their distribution, the dispersion index (σ^2^/y), and the *u* index.

**Table:**
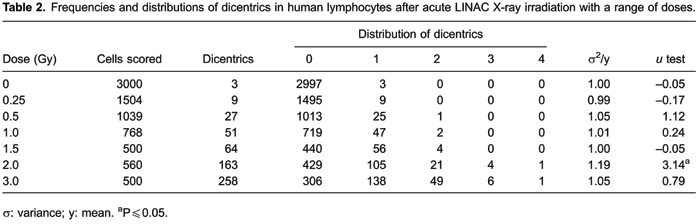

**Figure 1:**
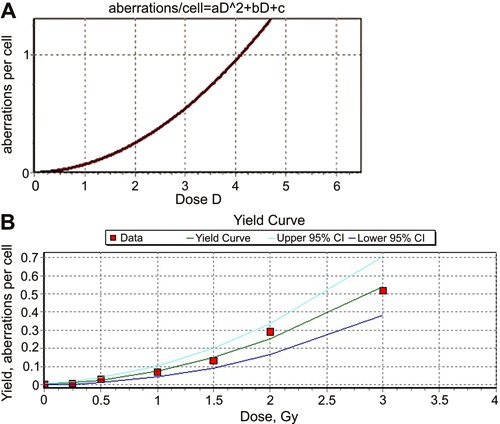
Dose-response calibration curve for dicentric yields induced by irradiation with LINAC X-rays. Data fitted with CABAS (*A*) and Dose Estimate (*B*) programs.

Figure 1 shows the dose-response calibration curves generated by the CABAS (A) and Dose Estimate (B) programs and calculated using the dicentric yields induced by incremental doses of X-irradiation generated by the linear accelerator. The curve fitted by the Dose Estimate program includes 95% CIs with upper and lower limits. The resultant α and β linear and quadratic yield parameters of the fitted curves and goodness-of-fit test results are shown in Table 3.

**Table:**
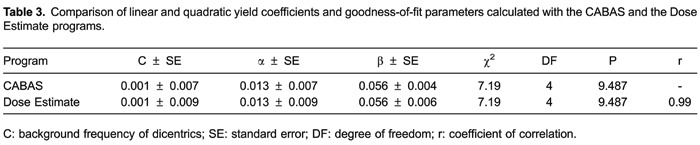

## Discussion

As expected, the yield of dicentrics increased with radiation dose, showing increments that were clearly dose-dependent (r=0.9680). Other chromosomal abnormalities observed (e.g., centric rings and excess fragments) also exhibited a dose-dependent response, but have been included only for completeness. They are not normally used in biodosimetry, and therefore we have not shown fitted curves for that data.

The yield of dicentrics for gamma and X-rays, which are both low with LET radiation, and where the ionizing events are sparsely distributed among cells, followed a Poisson distribution. This result is consistent with a random distribution of cellular and molecular damage (17,18). From Table 2, it can be seen that the dispersion index values were close to 1, while *u* values were between ±1.96, confirming that almost all data points were consistent with a Poisson distribution. However, for the 2 Gy dose, the dicentric distribution was significantly overdispersed. The overdispersion of data can be caused by nonhomogeneous irradiation of blood samples, resulting in nonuniformity of radiation effects. This is not uncommon for high doses, and has been reported by others for low LET radiation (2,18–22). In this study, the overdispersion can be accounted for by just one cell with four dicentrics.

In Figure 1, the coefficients of the dose-response curve were calculated using CABAS and Dose Estimate programs, which are based on the ML and IRLS methods, respectively. It has been pointed out that for data that have a truly Poisson distribution, the ML and IRLS methods should give the same results, with only slight differences observed in the standard errors of the α and β coefficients (7). This was certainly evident in this study where CABAS data fitted to Y=C+αD+βD^2^, where Y=(0.001±0.007)+(0.013±0.007)D+(0.056±0.004)D^2^; and the Dose Estimate data fitted to Y=(0.001±0.009)+(0.013±0.009)D+(0.056±0.006)D^2^.

In the present study, the goodness-of-fit test for the dicentric calibration curves indicated that the data were well represented by the linear-quadratic model (χ^2^=7.19, degrees of freedom=4, P=9.487). Moreover, values of correlation coefficients close to 1.0 (0.7 ≤ r <1) indicated a very strong relationship between the fitted data points.

As previously emphasized, the radiation-induced dicentric yield is also determined by the energy, so that it is interesting to compare the curves obtained for different types of radiation (4,5). With this intention, Table 4 shows the results of the present study compared with results obtained by other groups using different types of low LET radiation, i.e., 100-250 kVp X-rays and gamma-radiation (^60^Co and ^137^Cs). Comparing these different dose-response calibration curves, it is possible to notice a certain degree of variability in the fitted coefficients (α and β) for the different radiations.

**Table:**
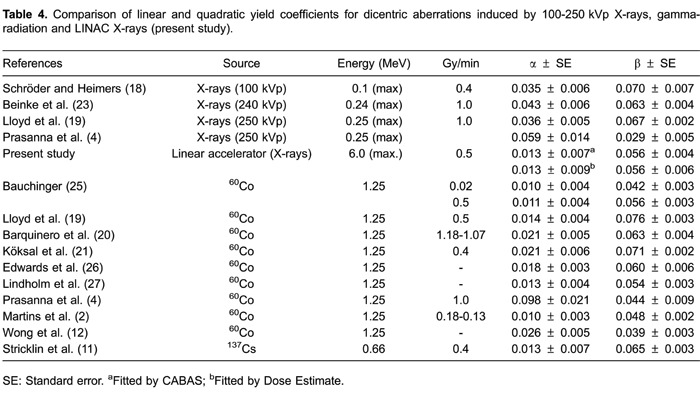

Several factors are known to have an impact on the resulting calibration curves, such as differences in the lymphocyte donors and culture protocols, slide preparation and scoring criteria. Therefore, to increase the accuracy of dose estimation, each laboratory should have its own calibration curve. Moreover, factors like the type of radiation, energy, and dose rate employed, all directly influence the values of α and β, considering the respective relative biological effectiveness (RBE) of different energies for producing dicentric chromosomes (5,11,20,23).

Even though X-ray radiation was used in the present study, it does not follow that the α and β parameters of our dose-response curve would be similar to those fitted for X-ray standard calibration curves fitted by Lloyd et al. (19), Schröder and Heimers (18), Prasanna et al. (4), and Beinke et al. (23) who all used orthovoltage X-rays.

Table 4 clearly illustrates how the linear coefficient is influenced by radiation quality, tending to be reduced at higher energies. This demonstrates very clearly how this biological assay endpoint has the ability to discriminate among differing relative effectiveness of low LET radiation, particularly at lower doses, which is regarded by the radiological protection community to have a weighting factor of 1.0. Indeed, fitted coefficients are more similar to α and β values obtained for the gamma-radiation curves (^60^Co and ^137^Cs sources), which have a higher energy than conventional X-rays.

Table 5 compares the data from this study with the other published calibration curves for LINAC X-rays. The coefficients were refitted with CABAS and Dose Estimate to ensure that the differences presented were not artifacts caused by the necessary assumptions and approximations of other curve-fitting programs.

**Table:**
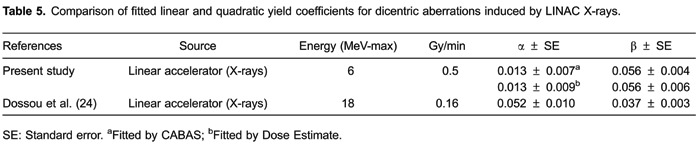

The data shown in Table 5 revealed an exception to the trend shown in Table 4of the α coefficient being increased at lower energies. In contrast to our present LINAC curves, the α coefficient of Dossou et al. (24), despite being the response to 18.0 MV, is higher and more similar to those found with orthovoltage X-rays rather than gamma sources. A high alpha term is consequently accompanied by a low β coefficient; this is sometimes referred to as the "see-saw" effect.

The beta term is influenced by the applied dose rate whereas the alpha term is LET dependent, thus the reason for this divergence in fitted coefficients probably lies in the dose rate used by investigators, since decreasing the dose rate changes the shape of the calibration curves as the linear coefficient tends to dominate (25–28). It arises from the increased time over which the irradiation is delivered, allowing time for DNA repair.

According to the multi-hit model, by prolonging the time of irradiation the chromosome break produced by the first track is already repaired when the second one crosses the cell, so that the chromosomes are unable to form an exchange aberration by nonhomologous end joining. Therefore, the dicentric frequency per unit dose is decreased at lower doses. This is particularly evident at higher doses where many more ionizing tracks cross the cell so that the likelihood of two-track exchanges is much greater and is described by a dose-squared term (βD^2^) (1).

It is generally accepted that biodosimetry laboratories should produce several curves to cover all the radiations likely to be involved in accidents. Considering the similarity between the values of fitted α and β coefficients in the present study at a dose-rate of 0.5 Gy/min of 6 MV LINAC, and those fitted for acute ^60^Co and ^137^Cs dose-response curves, one can conclude that some of these curves have a good biological equivalence.

According to Roch-Lefèvre et al. (29), the practical importance of knowing the parameters related to *in vitro*irradiation conditions is to determine long-term risks and hazardous effects of ionizing radiation on the health of exposed individuals. Those authors used biodosimetry in lymphocytes to assess the outcome of radiotherapy in patients treated with ^60^Co and LINACs using the same calibration curve. They found a strong correlation between the size of radiotherapy target field and the yield of radiation-induced chromosome aberrations, indicating that late toxic effects of radiotherapy might be determined by such cytogenetic assessment.

Given the similarity of the curve coefficients obtained in the present study with published values for gamma radiation, rather than orthovoltage X-rays, it follows that curves obtained for LINAC radiation have the potential to be used in radiotherapy as an additional means of quality control of the delivered absorbed dose and physical dosimetry. This curve may also be useful for *in vitro* dose reconstruction after accidental occupational exposures involving high energy X-rays, which is the basic recommendation in radiological emergency programs.

In conclusion, both the energy of the radiation and the dose rate are important considerations when constructing calibration curves for biodosimetry. Given the paucity of published LINAC X-ray data, additional study of the similarities and differences of these parameters, such as attempting to distinguish between the relative effects of energy versus dose rate, is necessary. The goodness-of-fit results for the dose-response calibration curves generated by CABAS and Dose Estimate software show that both programs can be used to investigate absorbed doses.
